# Outcome of Mears procedure for Sprengel’s deformity

**DOI:** 10.4103/0019-5413.77132

**Published:** 2011

**Authors:** Atul Rajeshwar Bhasker, Sachin Khullar, Mohamed Habeeb

**Affiliations:** Children Speciality Clinic, Mumbai, India; 1 Department of Orthopedics and Trauma, Indira Gandhi Memorial Hospital, Male’, Maldives

**Keywords:** Klippel Feil, Mears procedure, scapular osteotomy, Sprengel’s shoulder

## Abstract

**Background:**

Sprengel’s shoulder is characterized by scapular maldescent and malposition, causing restriction of shoulder and cervical spine movements. It is associated with a variety of other congenital anomalies. Various surgical procedures have been described to treat this anomaly with no consensus as to the surgical procedure of choice. We report the results of the Mears procedure in the treatment of Sprengel’s shoulder.

**Materials and Methods:**

Seven children between the age group of two and six years were treated for Sprengel’s deformity, with omovertebral bar, and other congenital anomalies. The Cavendish score and Rigault radiological score were used to assess the severity of the deformity, and the position of the scapula relative to the cervical spine, respectively. The Mears procedure involved scapular osteotomy, par tial scapular excision, and release of a long head of triceps. Clavicular osteotomy was done only in two cases to decrease the risk of traction injury to the brachial plexus. Postoperatively, the patients were immobilized in a shoulder sling and range of motion exercises were started as early as possible. The patients were followed regularly at six weeks, three months and regularly at six-months interval.

**Results:**

The mean improvement in flexion and abduction was 45 ° (40 – 70 °) and 50 ° (40 – 70 °), respectively, which was the combined glenohumeral and thoracoscapular movement. The cosmetic and functional improvement by this procedure was acceptable to the patients. Minor scar hypertrophy was seen in two cases.

**Conclusion:**

The Mears procedure gives excellent cosmetic and functional results. This procedure addresses the functional aspect of the deformity and is much more acceptable to the patient and parents.

Sprengel’s deformity is characterized by a high-riding scapula, asymmetry in the shoulder contour and restriction of shoulder movement. It is caused by a variable arrest in the descent of the scapula during intrauterine development.^1^,^2^ Sprengel’s deformity was first described by Eulenberg, in 1863, as, ‘hochgradige dislocation der scapula’ (i.e., a high-grade dislocation of the scapula),^3^ but it was Sprengel in 1891, who illustrated this deformity in four cases, and hence its name.^4^ –^6^ In 1883, Willet and Walsham were the first to describe the omovertebral bone and the methods of its excision.^7^ Depending on the severity, the deformity could be obvious at birth or manifest later in childhood. Occasionally Sprengel’s deformity could also occur as part of the Klippel Feil Syndrome (in 30% cases)^2^,^4^,^8^ or could be associated with other spinal and cranial anomalies^2^,^8^ or absent ribs.^9^ Several treatment options and techniques were described in literature that mainly focussed on positioning the scapula at its normal anatomical location.^6^,^10^,^11^ These had a limited success rate as Sprengel’s shoulder was a complex deformity and not merely an undescended scapula. In fact with those methods, recurrence of deformity, loss of function, and neurological problems were seen.^10^–^12^ In 2001, Dana Mears^13^ described a new surgical technique, which involved scapular osteotomy, partial excision of the scapula, and release of the long head of triceps, to improve the function of the shoulder. There is a paucity of information regarding the role of the Mears procedure in Sprengel’s deformity. To date only two series have been published in English literature using the Mears technique, of which one is by the original author.^13^,^14^ We report our experience with this technique in a small series of seven cases.

## MATERIALS AND METHODS

**Figure 1:**
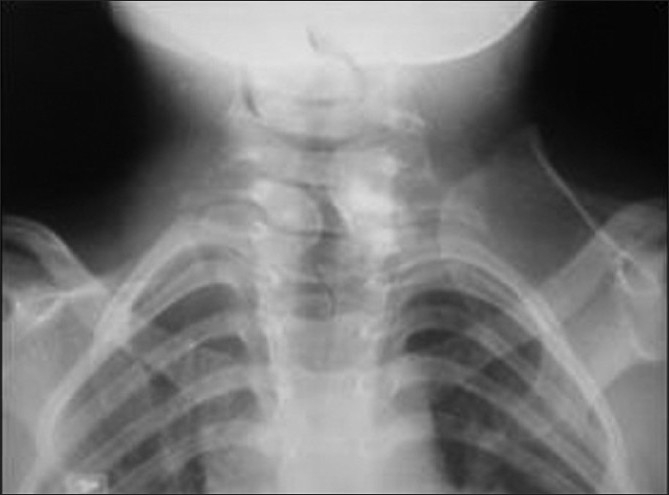
Preoperative X-ray chest showing high scapula on the left side with omovertebral mass

Seven children with Sprengel’s deformity operated between 2002 and 2006 were reviewed retrospectively in the Children Orthopedic Clinic. In our study, there were four girls and three boys, in the age group of two to six years. The left side was involved in four cases and in all the cases the right side was the dominant. The omovertebral bar was present in six cases. Ultrasound of the abdomen was performed in all cases to rule out other anomalies. Other congenital anomalies such as single kidney and Klippel-Feil syndrome were also observed in three cases. The parents’ main concern was the prominence due to malposition of the scapula and the restriction of the motion at shoulder. The children had complained of subjective pain on extreme abduction of the shoulder, due to the impingement. There was no neurovascular compromise in any child preoperatively [Table 1]. Preoperatively, all the cases were assessed clinically and radiologically. Radiographs of the chest and cervical spine were taken and the superomedial angle was taken as a reference to assess the scapular level and other anomalies [Figure 1]. The Cavendish score^15^ was used to grade the severity of the clinical deformity and the Rigault radiological score to assess the position of the scapula, relative to the cervical spine [Tables 2 and 3].

**Table 1:** Clinical details of patients

Case	Age	Sex	Side	Cg	Rg	Ob	Anomaly
1	2 y 2 m	M	Left	3	3	Present	KF
2	5 y	F	Left	3	2	Present	-
3	3 y 6 m	F	Left	3	3	Absent	One kidney
4	4 y	M	Right	3	2	Present	-
5	5 y	F	Left	2	2	Present	KF
5	5 y	F	Left	2	2	Present	-
7	6 y	F	Right	3	2	Present	-

y — Years, m — Months, CG — Cavendish grading, RG — Regault grading, OB — Omovertebral bar, KF — Klippel Feil syndrome, M — Male, F— Female

**Table 2:** Cavendish classification^15^

Grade 1	No visible deformity, patient fully dressed
Grade II	Bumpy aspect of the superomedial angle visible
Grade III	Shoulder asymmetry, 2 − 5 cm
Grade IV	Shoulder asymmetry, > 5 cm

This classification is difficult to apply in bilateral cases

**Table 3:** Radiographic classification (Rigault 1976)

Grade I	Superomedial angle lower than T2, but above T4
	transverse process
Grade II	Superomedial angle located between
	C5 and T2 TP
Grade III	Superomedial angle located above C5 TP

The parents of the children were counseled before the surgery and explained the expected outcome of the procedure, and informed consent for surgery was taken. All the children underwent the Mears procedure under general anesthesia, with the patient in a prone position. The high riding scapula was exposed by midline or curvilinear incision. The midline incision was used in two cases and curvilinear incision was used in five cases. The communication between the superomedial angle and the omovertebral bar was excised. As described oblique osteotomy through the body, along with sufficient resection of the scapula was done to avoid impingement. The long head of the triceps was released, to increase the abduction range. Clavicle osteotomy was done in two cases (Case Nos. 4 and 7), who presented after the age of four years, and had a risk of traction injury to the brachial plexus.

Postoperatively, the patient was immobilized in a shoulder sling and range of motion exercises were started when the child was pain-free (usually after two weeks). The patients were reviewed at six weeks, three months, and then regularly at six-month intervals.

## RESULTS

The mean follow-up was 2.4 years (2 – 3 years). On the affected side, preoperatively, the average flexion at the shoulder was 75°(50 – 100°) and the mean abduction at the shoulder was 85° (60 – 120°). The mean improvement in the flexion and abduction was 45° (40 – 70°) and 50° (40 – 70°), respectively. In this complex deformity of the shoulder girdle, it is very difficult to isolate the scapulothoracic motion from the glenohumeral movement, and therefore, the abduction recorded was a combination of glenohumeral and thoracoscapular movements and no physical methods were used to measure these movements separately. Although the children improved in their range of motion in other directions as well, we did not specifically measure the movements of extension, adduction, and rotation of the shoulder. Also the parents’ main concern was the inability to lift the arm as compared to the opposite side.

**Figure 2:**
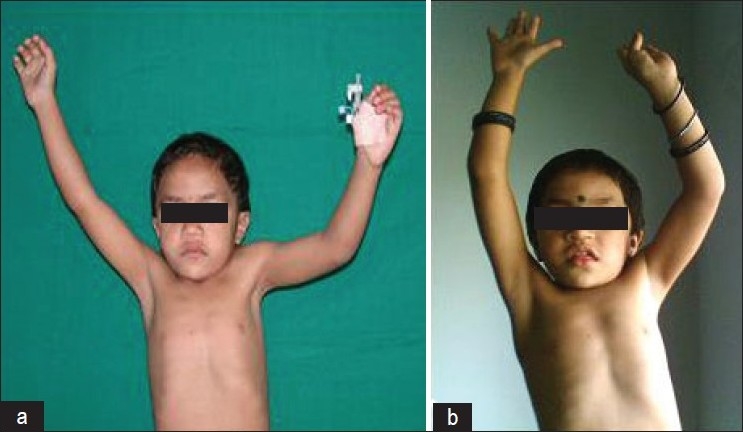
Clinical photograph showing (a) Preoperative range of abduction (b) Postoperative abduction: improved 40°;

**Figure 3:**
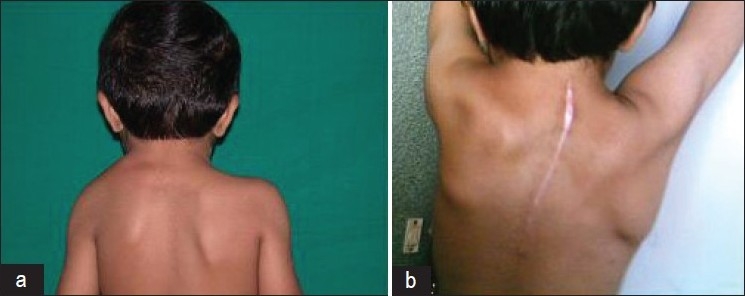
Clinical photograph showing (a) relative positions of the scapula, left scapula is higher (b) Operative scar: longitudinal incision

The range of motion improved gradually over three months and persisted till the final follow-up [Figure 2]. Minor scar hypertrophy was seen in two cases in children where the curvilinear incision was used [Figure 3]. No child complained of impingement pain, which was present preoperatively. The postoperative radiograph done at six months showed healing of the scapula osteotomy. The scapular size, however, remained small as compared to the contralateral normal side. Clinically, all the children had a muscle power comparable to the opposite side by six months, postoperatively. However, we had not used any mechanical device for measuring any muscle power. The improvement in various movements during the follow-up period is tabulated in Table 4.

**Table 4:** Functional results of patients

Case	Follow up (in months)	Abduction (in degrees)		Flexion (in degrees)		CG
		Pre op	Post op	Pre op	Post op	
1	24	60	120	50	90	1
2	18	100	160	90	140	1
3	36	110	150	70	120	1
4	18	120	160	90	130	1
5	26	80	130	100	160	1
6	28	90	160	100	150	1
7	26	110	150	90	160	1

Preop - Preoperative, Postop - Postoperative, CG - Cavendish grading

## DISCUSSION

In Sprengel’s deformity, the main problem is restricted motion of the shoulder and poor cosmesis. The different treatment modalities and surgical techniques described in literature have been as varied and complex as the deformity itself.

Woodward’s procedure^10^ has been considered the gold standard and the reference procedure, with over 80% satisfactory functional and cosmetic results. In the Woodward’s procedure, the muscles are resected extraperiosteally and sutured back (after lowering of the scapula to more inferior) to vertebral spinous processes. Younger patients obtain better motion and postoperative correction In the original Green’s procedure (Scapulopexy),^11^ muscle resection is done distally, rather than proximally. The muscles are reattached higher than the acromiothoracic junction’s rotation center. This procedure supposedly allows both lowering and rotation of the scapula, which provides a better biomechanical effect. In both the procedures, modification in the original procedure, in the form of clavicular osteotomy (Klisics modification), resection of the insertion of the supraspinatus muscle, and suturing of the inferior pole of the scapula to the thoracic cage into a pocket of the latissimus dorsi muscle (Leibovic’s modification) have been performed, to improve the results.

Andraults **et al**. in their study on eight children using the Greens method, found that in this procedure extensive dissection was required and the procedure was technically demanding.^11^ Leibovic **et al**.^5^ in their report in which they used the modified Green procedure to correct the Sprengel’s deformity, devised a radiographic geometric method, to quantitate the lowering and de-rotation of the scapula. The lowering did not change appreciably with time. The original malrotation of the scapula, which was corrected initially, recurred after two years. Doita **et al**.,^16^ showed good results after surgical correction in two adults using the Greens procedure, although surgical correction in older patients (> 8 years) still remains controversial.

Ross and Cruess,^17^ in their review of 77 cases, in which the surgical correction of congenital elevation of the scapula was done by the Woodwards procedure, Greens procedure, and Shrock’s procedure found that postoperatively, proximal resection increased shoulder abduction to 126°, but did not change the scapular position. Scapular relocation increased abduction to a mean of 134° and the shoulder position was altered from a mean of 1.8 inches of elevation, as compared to the normal side, to a mean of 0.5 inches of residual elevation. Significant loss of initial correction occurred in 14 out of 36 cases of proximal resection and 9 of 41 patients with scapular relocation.

In the original article published by Joe Woodward, the results were not entirely satisfactory. The improvement in the shoulder contour was offset due the hypertrophied scars, and transient brachial plexus injury was also observed.^8^ Despite the extensive muscle and soft tissue release in the above procedures, the results were not satisfactory.

Recent reports have highlighted good results with the Mears Technique. In the original procedure flexion improved from 100° to 175° and abduction improved from 90° to 150°. In one patient, a second operation was performed to remove an exostosis that followed the primary procedure. Initially, two keloid scars followed the use of a curvilinear incision. However, subsequently, this problem was eliminated by the use of a transverse incision.^13^

In another study by Dr. Javier **et al**., 14 patients with Sprengel shoulder were managed by the Mears procedure. In these patients, both flexion and abduction improved by more than 60°, with significant improvement in the range of motion. The appearance improved in all the patients. Two cases of keloid formation were seen. It has been well-documented in their study that there is no correlation between the position of the scapula and the amount of lowering with the final outcome of the procedure.^14^

We performed clavicular osteotomy in two cases, where the children who presented were over four years of age, with Cavendish grade 3 (Case Nos. 4 and 7), to offset any potential risk of injury to the brachial plexus. Mears and Javier **et al**. did not describe any clavicular osteotomy in their series of cases and hence we also felt that it might not be required. A keloid scar was seen in two cases in the medial part of the curvilinear incision. Hence, we felt that a midline longitudinal or transverse incision might be cosmetically more superior.

Postoperatively, we deferred active mobilization of the shoulder until wound healing, as perioperative analgesia facilities were suboptimal in our setup. We did not want to aggravate postoperative pain and increase the patient’s apprehension. However, this did not affect the functional outcome of the procedure. The limitation of our study was that this was a small series of only seven cases with a small follow-up. However, our results are comparable to the already published series.

The Mears procedure directly addresses the functional and cosmetic aspects and is one of the good option to treat Sprengel deformity.
